# New Eco-Cements Made with Marabou Weed Biomass Ash

**DOI:** 10.3390/ma17205012

**Published:** 2024-10-14

**Authors:** Moisés Frías, Ana María Moreno de los Reyes, Ernesto Villar-Cociña, Rosario García, Raquel Vigil de la Villa, Milica Vidak Vasić

**Affiliations:** 1 Eduardo Torroja Institute for Construction Sciences, IETcc-CSIC, 28033 Madrid, Spain; 2 Department of Physics, Universidad Central de las Villas, Santa Clara 54830, Cuba; ernestovillarcocina@gmail.com; 3 CSIC-Affiliated Geomaterials Unit, Department of Geology and Geochemistry, Autonomous University of Madrid, 28049 Madrid, Spain; rosario.garcia@uam.es (R.G.); vigildelavillaraquel@gmail.com (R.V.d.l.V.); 4 Institute for Testing of Materials IMS, 11000 Belgrade, Serbia; milica.vasic@institutims.rs

**Keywords:** Marabou weed biomass ash, mineralogical addition, binary eco-cement, electrical resistivity

## Abstract

Biomass ash is currently attracting the attention of science and industry as an inexhaustible eco-friendly alternative to pozzolans traditionally used in commercial cement manufacture (fly ash, silica fume, natural/calcined pozzolan). This paper explores a new line of research into Marabou weed ash (MA), an alternative to better-known conventional agro-industry waste materials (rice husk, bagasse cane, bamboo, forest waste, etc.) produced in Cuba from an invasive plant harvested as biomass for bioenergy production. The study entailed full characterization of MA using a variety of instrumental techniques, analysis of pozzolanic reactivity in the pozzolan/lime system, and, finally its influence on the physical and mechanical properties of binary pastes and mortars containing 10% and 20% MA replacement content. The results indicate that MA has a very low acid oxide content and a high loss on ignition (30%) and K_2_O content (6.9%), which produces medium–low pozzolanic activity. Despite an observed increase in the blended mortars’ total and capillary water absorption capacity and electrical resistivity and a loss in mechanical strength approximately equivalent to the replacement percentage, the 10% and 20% MA blended cements meet the regulatory chemical, physical, and mechanical requirements specified. Marabou weed ash is therefore a viable future supplementary cementitious material.

## 1. Introduction

One of the five key elements of the cement industry’s roadmap to achieving climate neutrality by 2050 is the 5C cement challenge to develop future commercial products with a lower clinker-to-cement ratio (≤0.60) than those currently manufactured. According to the data available as of 2021, clinker content in the Spanish cement industry stands at around 0.8 (clinker/cement ratio), a high factor exacerbated by the economic crisis in the sector, COVID-19, lower demand for cement, and lower availability of traditional additives [1,2]. This combination makes it imperative to continue the search for new supplementary cementitious materials (SCMs) that offer an eco-friendly alternative to those traditionally used (fly ash, silica fume, and natural/calcined clay) [3]. In recent years, the scientific community has conducted ongoing research into the different industrial waste streams that, due to their chemical, mineralogical, and pozzolanic characteristics, offer scientifically, technically, and environmentally viable substitutes [4,5,6,7,8,9,10]. Within these emerging streams, ash from biomass combustion is attracting particular attention because of the vast volumes generated globally (170 Mt/yr) [11], mainly driven by the new international scenarios promoting the use of clean energy from renewable sources [12]. There is a wide variety of biomass ash rich in reactive silica and alumina (rice husk, bagasse cane, bamboo leaf, and paper sludge, among others) capable of providing cement with the improved properties required by the transition to a low-carbon economy [13,14,15,16,17]. However, biomass ash has several drawbacks, such as its heterogeneity (time of year generated, energy process, burning temperature, etc.) and its contribution of potentially negative elements/oxides to the cement matrix. The latter is the case with high-potash (K_2_O) ash, which requires pre-washing to reduce its content and prevent reactivity and durability issues (alkali-aggregate reaction) in the new eco-cements [18,19,20,21,22].

Intending to expand the range of biomass ashes potentially viable as eco-pozzolans, this paper analyzes new ash obtained from the combustion of Marabou weed (harvested in Cuba). To this end, the study entails full characterization using a variety of instrumental techniques and analysis of pozzolanic reactivity and its influence on the physical and mechanical properties of binary eco-cements made with 10% and 20% replacement content used in the manufacture of CEM II-A cement.

## 2. Materials and Methods

### 2.1. Materials

**Figure 1:**
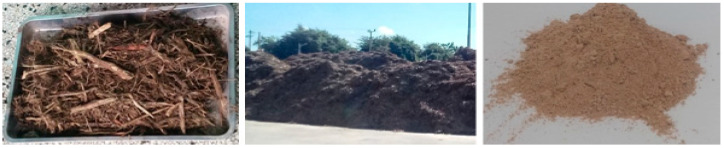
The appearance of Marabou weed before and after thermal processing.

Marabou (MA) weed (Dichrostachys cinerea) is a semi-deciduous shrub from the legume family. It is native to South Africa and can reach 7 m in height. In Cuba, the plant is estimated to grow on more than 1.2 million hectares at an average density of 37 t/ha [23,24,25]. It is considered an invasive species and an important alternative fuel for biopower stations. In 2016, Cuba produced 154 GWh of bioenergy from Marabou weed biomass [8]. Since Marabou weed harvesting is seasonal, the biopower plant did not use it as fuel at the sampling time. The ash (MA) was therefore produced at a laboratory scale, by calcination to 600 °C for 2 h in a muffle furnace, considered optimal conditions from a pozzolanic, energy, and economic point of view (Figure 1). In addition, carbonates will not degrade at this temperature, although organic matter will be completely decomposed.

In this study, a commercial CEM I 52.5R-type cement (OPC) complying with European standard EN 197-1 [3] and supplied by Cementos Lemona, S.A. (Bilbao, Spain) was used. The binary cements were made by replacing part of the cement with 10% and 20% MA to obtain CEM II-A (6–20%) cements. Standardized commercial sand at a cement/sand ratio of 1:3 and a *w*/*c* ratio of 0.5 was used to manufacture the mortars.

### 2.2. Experimental Methodology

#### 2.2.1. Pozzolanic Activity Method

To assess the pozzolanic activity of the MA, the accelerated chemical method was applied to the pozzolan/lime system [8]. The test consisted of adding 1 g of pozzolan to 75 mL of saturated lime solution kept in a laboratory stove at 40 °C for 7, 28, and 90 days of reaction. At the end of each period, the solid obtained was filtered and washed with ethanol and then dried in a stove at 60 °C for 24 h to halt the pozzolanic reaction. The fixed lime values were used to generate the diffusive kinetic model [26], as per Equation (1):

(1) $$C_{t}=\frac{0.23\cdot exp\left(\frac{-3t}{\tau }\right)\cdot \left(-1+exp\left(\frac{t}{\tau }\right)\right)\cdot \frac{1}{\tau }}{D_{e}\cdot r_{s}}+\frac{0.23\cdot exp\left(-\frac{1}{\tau }\right)\cdot \frac{1}{\tau }}{K\cdot r_{s}^{2}}+C_{corr}$$

where D_e_ is the effective diffusion coefficient, K is the reaction rate constant, τ is the time constant (the time interval during which the pozzolan radius diminishes to 37% of its initial radio r_s_), C_t_ is the absolute loss of CH concentration with time for the pozzolan/lime system, and C_corr_ is the correction parameter that takes into account the concentration remainder of CH that is not consumed in the reaction. Depending on the pozzolanic reaction, the behavior is as follows: diffusive (described by the first term), kinetic (described by the second term), and kinetic–diffusive (both terms).

#### 2.2.2. Rheological Properties

The water required for normal paste consistency (NPC), the initial setting time (IST), and the volume stability (S) of the fresh cement pastes were determined as per EN 196-3 [27].

#### 2.2.3. Physical Properties

The capillary absorption capacity of the mortars was analyzed in prismatic specimens measuring 4 × 4 × 16 cm and previously cured for 28 days using the Fagerlund method, as described in UNE 83982 [28]. Upon completion of the curing phase, the specimens were conditioned as per Spanish standard UNE 83966 [29] to obtain homogeneous moisture distribution throughout them. After conditioning, the specimens were placed in a container and partially immersed in 5 mm of water. The capillary absorption coefficient (K) in $kg\cdot m^{2}\cdot {min}^{0.5},$ effective porosity (*ε*) in cm^3^·cm^−3^, and resistance to water penetration by capillary absorption (m) in min·cm^−2^ were determined by applying Equations (2)–(4), respectively:

(2) $$K=\delta^{a}\cdot \varepsilon_{e}/10\cdot \sqrt{m}$$

(3) $$\varepsilon_{e}=Q_{n}-Q_{0}/A\cdot h\cdot \delta_{a}$$

(4) $$m=t_{n}/h^{2}$$

where δ_a_ is the density of the water (considering the value of 1 g·cm^−3^), Q_n_ is the weight of the specimen at saturation (t = t_n_), Q_0_ is the weight of the specimen before the test (t = 0), A is the section of the specimen, h is the thickness of the specimen, and t_n_ is the period required to reach saturation.

Analysis of electrical resistivity in the mortars was performed on prismatic specimens measuring 4 × 4 × 16 cm saturated with water for up to 90 days of curing. To this end, the 4-electrode Wenner method, as described in Spanish standard UNE 83988-2 [30], was used. Resistivity (ρ) was calculated by applying Equation (5):

(5) $$\rho =\rho_{w}\cdot F_{f}$$

where F_f_ is the form factor (which amounts to 0.172 for samples measuring 4 × 4 × 16 cm) and $\rho_{w}$ is the Wenner resistivity. The age factor (q) [21,31] meanwhile describes the changes in resistivity over time and adjusts the resistivity curve over time through Equation (6):

(6) $$\rho_{t}=\rho_{0}{\left(t/t_{0}\right)}^{q}$$

where $\rho_{t}$ represents the resistivity measured at time t, and $\rho_{0}$ represents the resistivity at time 0 (t_0_).

#### 2.2.4. Mechanical Properties

The mechanical flexural and compressive strength tests performed on the mortars were conducted at 2, 28, and 90 days of curing, as per European standard EN 196-1 [32]. The loading speed used for the flexural strength test was 50 N·s^−1^, and the loading speed used for the compressive strength test was 2400 N·s^−1^

### 2.3. Instrumental Techniques

Chemical quantification was performed using a Bruker S8 Tiger wavelength dispersive X-ray fluorescence (WDXRF) spectrometer. Particle size distribution was obtained using a Malvern Mastersizer 3000 laser diffraction device equipped with red and blue light sources (He-Ne and LED) operating in dry dispersion mode. Measurements were taken in the range of 0.01–3500 μm. Identification and quantification of crystalline mineralogical phases (XRD–Rietveld method) were carried out with a PANalytical X’Pert PRO diffractometer, utilizing the Crystallography Open Database (COD) collection of crystal structures, and quantified using the Match v.3 and Fullprof software v.23. The morphology and element microanalyses were performed using an Inspect (FEI Company, Hillsboro, OR, USA) scanning electron microscope (SEM) equipped with an energy-dispersive X-ray (EDX) analyzer and a Si/Li detector. The pore size distribution and total porosity in the mortars were analyzed using a mercury intrusion porosimeter (MIP; Micromeritics Model 9320) in microspecimens measuring approximately 1 cm^3^.

The changes in heating curve and heat of hydration in the standardized mortars were obtained using the Langavant semi-adiabatic method set out in European standard EN 196-9 [33] employing an Ibertest IB32-101E and the WinLect32.06 software.

## 3. Results

### 3.1. Characterization of the Starting Materials

XRF Analysis

The chemical compositions of the OPC and the MA are shown in Table 1. The MA is calcic (45.42% calcium content), and the sum of its SiO_2_ + Al_2_O_3_ + Fe_2_O_3_ (4.43%) oxides is far below the level (≥70%) specified in the ASTM 618 standard for artificial pozzolans (fly ash and calcined natural pozzolan). The loss on ignition (LOI) value for MA (30%) is also above the standard limit (≤10%) [34]. The 6.92% K_2_O content is notable.

**Figure 2:**
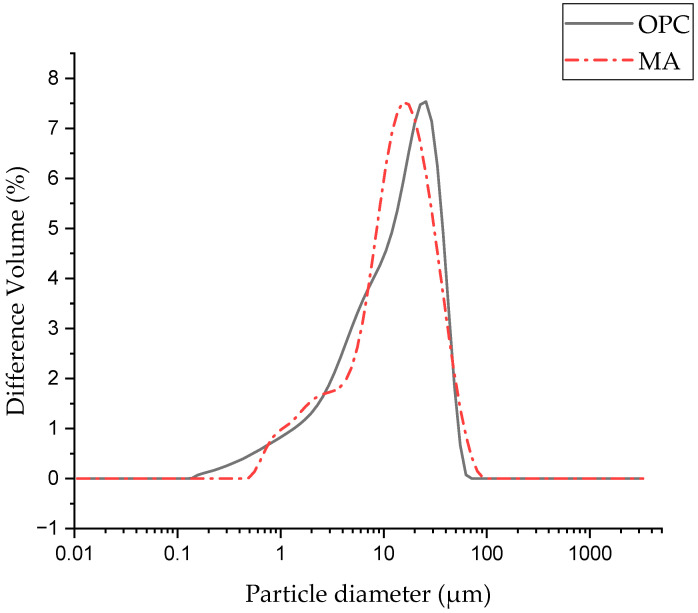
Density distribution curves of the starting materials.

The particle size distribution density curves for the two starting materials are shown in Figure 2. These materials show similar distribution densities, with the main peaks at 25.68 and 15.41 μm, respectively. This similarity is corroborated by the Dx values shown in Table 2.

Mineralogical quantification using the XRD–Rietveld method (Table 3) identifies anhydrous phases typical of OPC (C_3_S, C_2_S, C_3_A, C_4_AF, and CaCO_3_), while the MA mostly comprises quartz, calcite, and dolomite.

### 3.2. Pozzolanic Activity

#### 3.2.1. Accelerated Chemical Method

**Figure 3:**
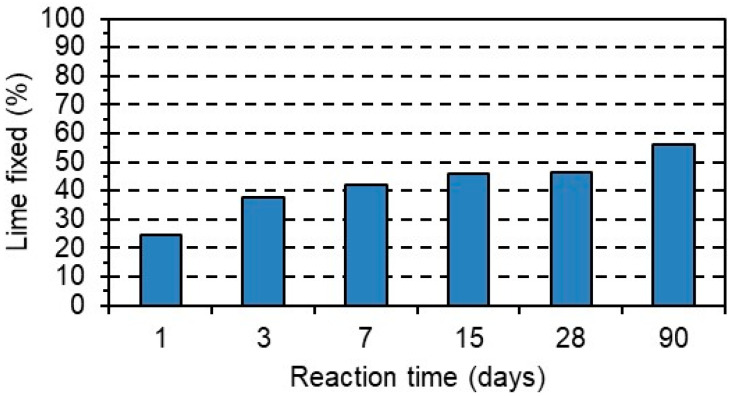
Changes in the amount of fixed lime with reaction time.

The values obtained up to 90 days of reaction (Figure 3) show that the MA exhibits low–medium pozzolanic behavior since at 90 d it has only consumed 55% of the available lime. This fact is related to the low levels of silica and alumina in the ash (3.65%). Furthermore, the fixed lime values might be slightly overestimated if the possible effect of potash (K_2_O = 6.92%) is taken into account, since it increases the pH of the solution and insolubilizes the portlandite, removing it from the medium [35].

Applying the fixed lime values set out in Equation (1) quantifies the corresponding parameters shown in Table 4.

The K(10^−3^) value obtained for the MA is of a lower order than other eco-pozzolans (silica fume, fly ash, natural pozzolan, and bagasse ash) [36].

#### 3.2.2. Mineralogical Analysis of the Pozzolanic Reaction Using the XRD–Rietveld Method and SEM–EDX

**Figure 4:**
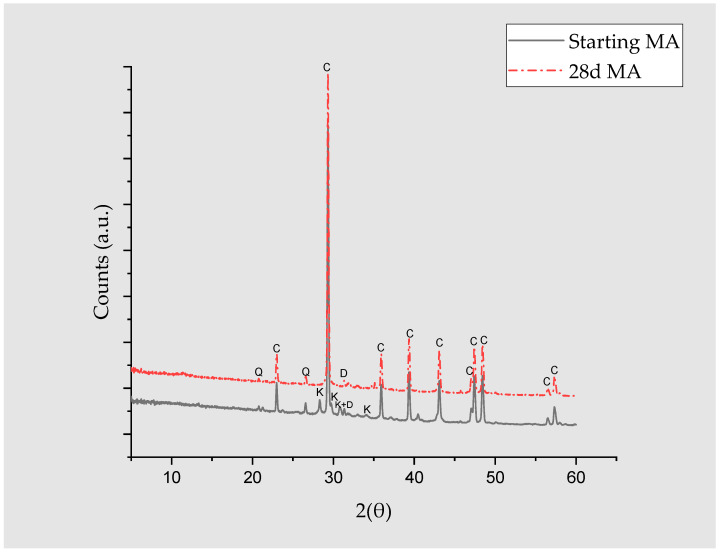
XRD diffractograms of the ash before and after 28 d. (C: calcite, Q: Quartz, K: Arkanite, and D: Dolomite).

After 28 days of reaction, the product presented no mineralogical differences compared to the starting MA (Figure 4), with quartz, calcite, and dolomite identified in both spectra (Table 5). The disappearance of the crystalline dolomite phase and the slight decrease in the amorphous phase due to the pozzolanic reaction are noteworthy. The calcite does not appear to intervene in the pozzolanic reaction, and its increase may be due to the carbonation of the sample during later testing, storage, and analysis.

**Figure 5:**
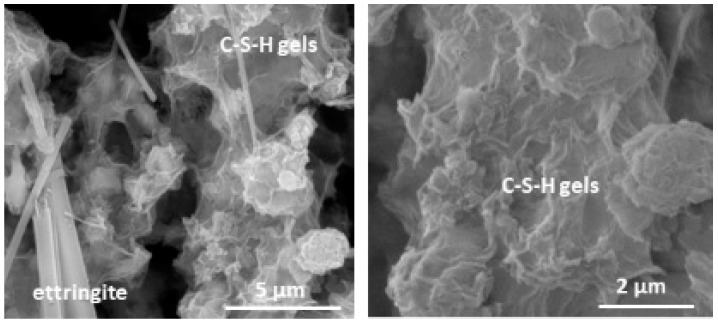
Ettringite and CSH gels (**left**) and detailed view of CSH gels (**right**).

SEM/EDX observation at 28 days of curing (28 d MA) identifies the formation of CSH gels (C/S = 2.33) and ettringite as products of the pozzolanic reaction (Figure 5 and Table 6).

### 3.3. Chemical Characterization of the Anhydrous Blended Cements

The addition of MA modifies the content of the major oxides in the blended cement (Table 7), decreasing the percentages of SiO_2_, Al_2_O_3,_ and CaO and increasing the share of potash and LOI. The blended cement containing 10% and 20% MA meets the chemical requirements (SO_3_ and Cl^−^) set out in EN 197-1 (SO_3_ ≤ 3.5–4.0% and Cl ≤ 0.1%) [3]. Meanwhile, the EN 450 standard [37] on the addition of fly ash to concrete limits the equivalent Na_2_O content (Na_2_O + 0.66 K_2_O) to 5%, a value well above that obtained in this study (1.23% and 1.65%, respectively).

### 3.4. Rheological Behavior of the Blended Cement Pastes

As per standard EN 196-3, normal consistency water (NCW), initial setting time (IST), and soundness (S) were analyzed (Table 8).

The addition of MA to the cement causes a slight increase in the NCW values versus the OPC (of 0.7% and 2.6%, respectively) due to the greater specific surface area of the ash. It also slightly accelerates the IST [38], albeit within the deviation range of the method. The blended pastes do not experience any expansion effect or similar behavior to the OPC paste.

Based on these results, the types of cement analyzed meet the standardized physical specifications for the manufacture of future commercial cement.

### 3.5. Physical and Mechanical Behavior of the Blended Cement Mortars

#### 3.5.1. Calorimetric Behavior of the Mortars

**Figure 6:**
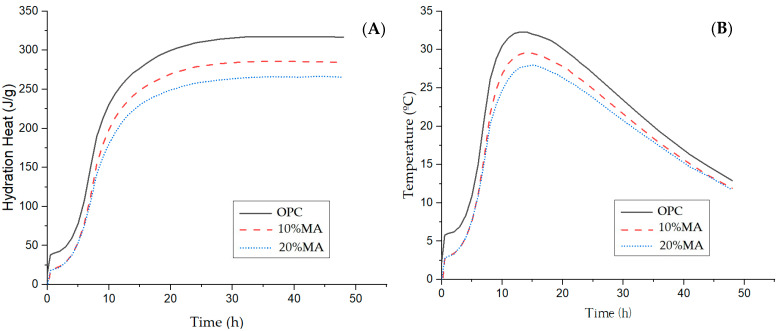
Langavant calorimetry: (**A**) heating curve and (**B**) hydration heat of the mortars.

The blended mortars containing MA present a different heating curve to the OPC mortar (Figure 6), showing lower maximum values of 29.6 °C (10% MA) and 27.8 °C (20% MA), respectively, versus 32 °C (OPC). In addition, this maximum value is recorded at greater reaction times—from 13 h (OPC) to 14 h—for the blended cement mortars [39,40]. These heating curve variations are reflected in the hydration heat values (Figure 6B), which decrease as the MA admixture percentage rises. Based on the data in Table 9 and the specifications in EN 197-1 [3], only the cement with 20% replacement content is considered a low heat-of-hydration cement since the value of 265.5 J/g is below the regulatory limit (≤270 J/g).

#### 3.5.2. Total and Capillary Water Absorption

Table 10 shows that the mortars containing MA experience an increase in water absorption capacity as the additive proportion grows, rising from 4.26% (OPC) to 5.63% for the 20% MA mortar, and an increase in absorption rate from 0.78 g/min^0.5^ to 0.94 g/min^0.5^. Similar results were obtained by other authors with ceramic waste, coal mining waste [41] and biomass ash [42]. Nevertheless, total absorption in MA cement mortars was below the 10% recommended for high-quality cement-based materials [43,44].

**Figure 7:**
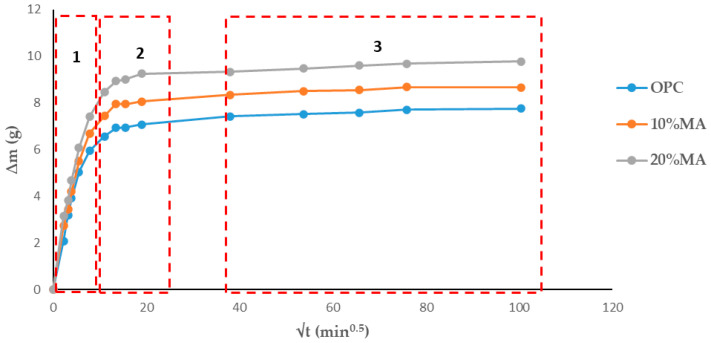
Total absorption curves in the mortars analyzed.

Table 11 shows the absorption rates obtained from the regression lines of Figure 7. An increase in the water absorption rate in the mortars containing MA is observed in the first and second absorption intervals (5 min–1 h) and (2–6 h), respectively, while a subsequent decrease versus the standard mortar is observed in the third interval (>6 h). The percentage of MA replacement content (10% and 20%) has little influence on the rate. This decrease in absorption rate is related to microstructural changes in the mortars studied due to the formation of hydrated phases, principally CSH gels [45].

**Figure 8:**
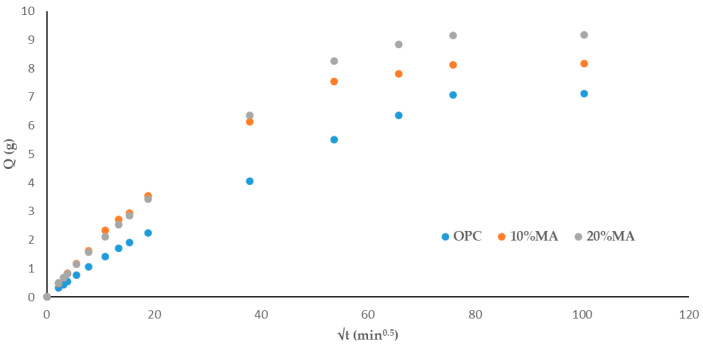
Capillary water absorption in the mortars.

The capillary absorption tests (Figure 8) show the same trend as for total water absorption: the higher the MA percentage in the cement, the greater the capillary water absorption capacity [46]. Two behaviors are observed: the first (0–20 min^0.5^) consists of water absorption via the capillary pore network, while the second (20–100 min^0.5^) corresponds to the filling of air pores via the air diffusion and dissolution process until saturation state is reached [47].

Applying the Fagerlund method [8] and employing Equations (2)–(4) (Table 12) allow for the determination of the capillary absorption coefficient (K), effective porosity ($\varepsilon_{e}$), and resistance to water penetration by capillary absorption (m). The 10% and 20% MA mortars have a higher absorption coefficient than the OPC mortar. This is attributed to the higher density of the OPC mortar and to the sealing of the pore network, which contributes to a reduction in capillary porosity versus the blended cement mortars. These results are in line with those obtained previously (Table 11) and those obtained with other cements [48,49], but not with the findings reported in [50]. As regards the m-coefficient (Table 12), an increase in the effective porosity values is observed with the addition of MA and is very similar in all three cases to that of the OPC, indicating interconnection between capillary pores in the mortars [51]. In addition, the $\varepsilon_{e}$-coefficient (Table 12), classified according to the concrete’s durability to penetration by aggressive agents as established by the CyTED RED DURAR [52,53] (where <10% indicates that the concrete is of good quality and compactness, 10–15% indicates moderate quality, and >15% indicates inadequate durability), shows that the values for the mortars studied are <10%. Taking into account the above, it can therefore be deduced that they are durable and of good quality [51].

#### 3.5.3. Electrical Resistivity

**Figure 9:**
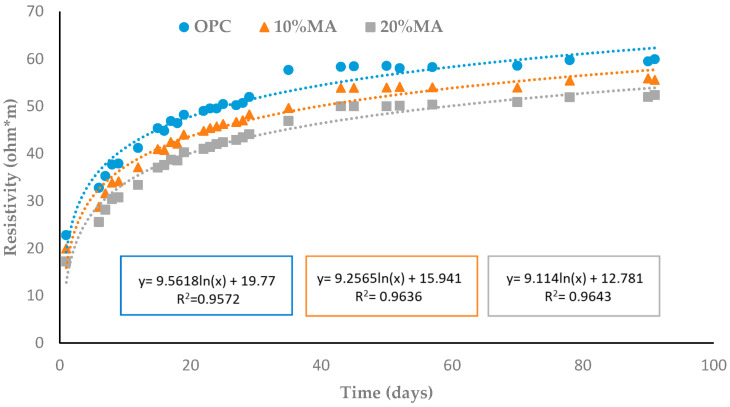
Changes in electrical resistivity as a function of hydration time.

The electrical resistivity results show an increase with the curing time in all the mortars (Figure 9). At 90 d, the MA cement mortars present lower resistivity values than the OPC (59.5 Ω·m) which decreases as the additive percentage rises (55.9 and 51.9 Ω·m, respectively). This fact is likely related to the addition of MA lowering densification of the matrix, thus offering less resistance to the electrical current [54,55,56].

The loss of resistivity observed in this paper is associated with content heterogeneity, especially as regards the presence of alkalis [57]—partly mirrors the findings of previous research into biomass ashes. The lower resistivity of the mortars containing MA is in line with the values obtained for the age factor (q) when applying Equation (5) (Section 2.2.3). The results corresponding to the age (q) and resistivity factors at time 0 ($\rho_{0}$) as per Equation (6), as well as the R^2^ values of the regression, are shown in Table 13. The q-values increase significantly as the percentage of ash rises from 0.23 in the OPC to 0.27 in the 20% MA. These results are in line with those obtained for capillary absorption (Section 3.5.2) and all these q-values follow the reference values [58] for mortars prepared with CEM I and CEM II/A-P (0.22 and 0.37, respectively).

#### 3.5.4. Compressive Strength and Microporosity

**Figure 10:**
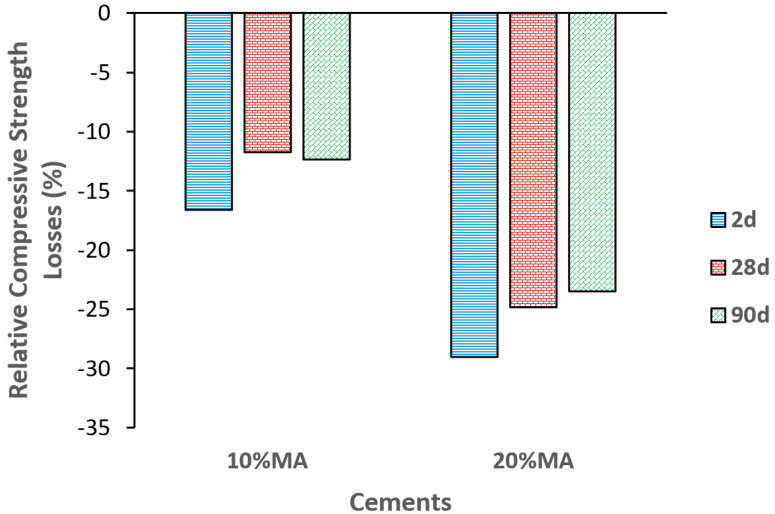
Changes in compressive strength losses versus the OPC mortar.

Analysis of the relative losses in compressive strength versus the OPC mortar (Figure 10) reveals, in general, that the MA additive reduces strength in proportions greater than the percentage of replacement content, i.e., by approximately 17% and 29%, respectively, at 2 d of hydration. At 28 and 90 d, no noticeable mechanical differences are observed, with relative losses of around 11% and 23% for the 10% and 20% MA mortars, respectively.

This decrease is related to the low pozzolanic activity, greater water absorption, lower density, and lower electrical resistivity in the MA mortars, as discussed above. Furthermore, the incorporation from the ash of additional K_2_O, known for its negative effect on the previously discussed hydration and pozzolanic reactions, should be taken into account. Thus, adding MA would mostly have a filler effect on the cement matrix. According to Table 14 and the mechanical requirements set out in EN 197-1 [3], MA blended cement maintains the initial strength category at 2 days, while at 28 days only the 20% MA cement would drop a strength category to 42.5.

**Figure 11:**
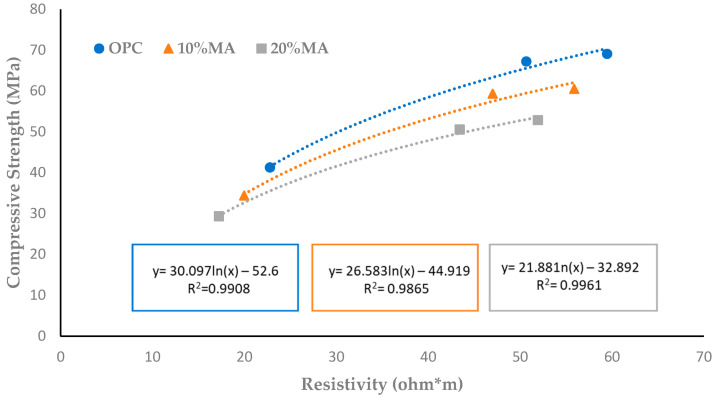
The relationship between compressive strength and resistivity.

An excellent correlation coefficient is found between the compressive strength and electrical resistivity values (R^2^ ≥ 0.98) (Figure 11) which, under standard conditions, makes it possible to predict one of these parameters based on the other [58].

**Figure 12:**
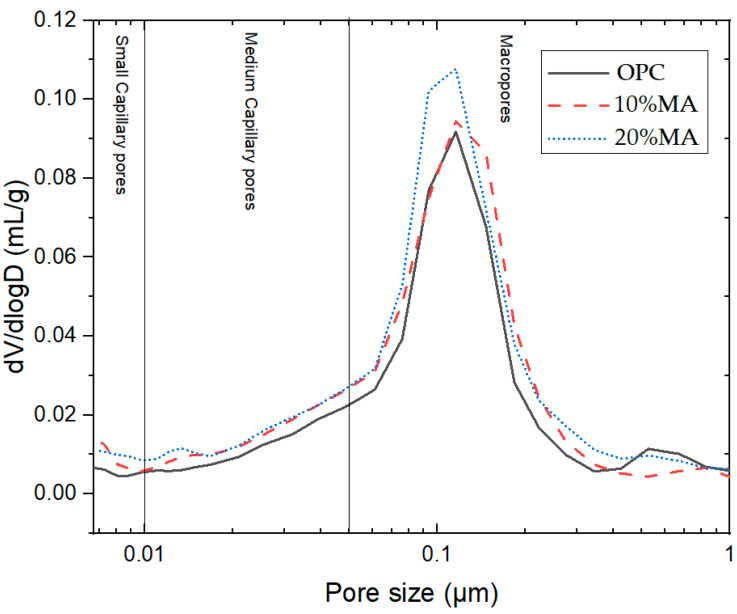
Pore distribution density curves at 90 d.

The total porosity of the mortars (Table 15) shows that the addition of 10% and 20% MA produces an increase in porosity versus the OPC of between 21% and 23% and of between 11% and 21% at 2 and 90 d, respectively. However, the shapes of the pore size density curves at 90 d (Figure 12) are very similar, showing a single peak between 60 nm and 200 nm, albeit with a slight refinement of the pore sizes below 100 nm when MA is added. This phenomenon is related to the low pozzolanic activity of MA, which does not compensate for the cement replacement effect [59]. This behavior is in line with other industrial waste materials presenting low–medium pozzolanic activity [60].

## 4. Conclusions

This work presents the quality of cement and mortar by replacing OPC with Marabou weed ash (MA) from Cuba. The following conclusions are drawn from the results obtained with MA:

1. Chemical analysis determined by the XRF of the MA shows that the sum of SiO_2_ + Al_2_O_3_ + Fe_2_O_3_ (4.43%) oxides is far below the minimum value recommended by the regulations for active cement admixtures (≥70%). In addition, it presents 30% LOI due to its calcium and unburned material content, and 6.92% K_2_O.
2. From a mineralogical point of view, MA comprises 46% calcareous minerals (calcite and dolomite) and 41% amorphous phase.
3. MA exhibits medium–low pozzolanic activity in the pozzolan/lime system due to its chemical and mineral composition. SEM-EDX analyses at 28 days of reaction identified CSH gels and ettringite as mineralogical phases produced by the pozzolanic reaction.
4. 10% and 20% MA blended cements meet the chemical (SO_3_ and Cl^−^) and physical (NCW, IST, and S) requirements set out in the regulations.
5. The heating curves and heat of hydration of the mortars containing 10% and 20% MA decrease in inverse proportion to the ash content. The types of cement made with 20% MA qualify as low heat-of-hydration cement (≤270 J/g).
6. In terms of intrinsic properties, the addition of MA results in an increase in water absorption (total and capillary), lower electrical resistivity (up to 13%), and classification of the blended mortars as durable from the point of view of penetration by aggressive agents.
7. Although, when compared to the OPC mortar, the MA cement mortars experience a decrease in compressive strength at 28 d of curing roughly equivalent to the replacement rate, the binary cement made with 10% and 20% MA meet the mechanical requirements for the manufacture of commercial cement. At 2 d of hydration, the materials maintain the initial strength category (52.5), but at 28 days the 20% MA cement drops to the lower strength category (42.5). A good coefficient of correlation (≥0.98) was found between strength and resistivity.
8. The porosimetry tests corroborate the findings of the intrinsic tests, highlighting that the MA cement admixture produces an increase in total porosity, although a slight refinement of less than 100 nm is observed in the pore density curves.

Based on the results obtained, Marabou weed biomass ash is viable as a mineralogical additive for use in the future manufacture of eco-friendly cement with a reduced carbon footprint. The findings of this pioneering study of MA will form the basis of future lines of research principally exploring higher replacement content percentages, synergies with other pozzolans, durability when subject to aggressive agents, etc.

## Figures and Tables

**Table 1:** Chemical composition of the OPC and MA, expressed in %.

	OPC	MA
SiO_2_	20.06	2.63
Al_2_O_3_	4.96	1.02
Fe_2_O_3_	3.28	0.78
CaO	62.19	45.42
MgO	2.12	6.36
SO_3_	3.14	4.14
Na_2_O	0.37	0.48
K_2_O	0.66	6.92
TiO_2_	0.25	0.09
P_2_O_5_	0.29	1.13
Cl	0.07	0.77
ZnO	0.17	-
SrO	0.16	-
Cr_2_O_3_	0.04	-
LOI	2.11	30.00

**Table 2:** Dx values for the OPC and MA.

Raw Material	D_10_ (µm)	D_50_ (µm)	D_90_ (µm)
OPC	2.11	14.10	34.40
MA	2.40	13.60	34.70

**Table 3:** Mineralogical phases were quantified using the XRD–Rietveld method (n.d. = not detected; R_B_ and X^2^ = adjustment factors).

Mineral (%)	OPC	MA
Quartz	n.d.	13
Calcite	4	34
Dolomite	n.d.	12
K-feldspar	n.d.	n.d.
C_3_S	52	n.d.
C_2_S	20	n.d.
C_4_AF	6	n.d.
C_3_A	9	n.d.
Amorphous matter	9	41
R_B_	17.2	19.6
X^2^	7.3	9.4

**Table 4:** Kinetic parameters of the pozzolanic reaction.

Parameters	$\tau \left(h\right)$	Rate K (h^−1^)	Free Energy of Activation AG^#^, kJ/mol	C_corr_	Coefficient of Multiple Determination (R^2^)
MA	108.9 ± 0.1	(2.57 ± 0.07)·10^−3^	113.61	0.88 ± 0.007	0.9852

**Table 5:** Analysis using the XRD–Rietveld method.

%	Quartz	Calcite	Dolomite	Amorphous Phase	R_B_	X^2^
Starting MA	13	34	12	41	19.6	9.4
28 d MA	16	47	traces	37	18.6	7.4

**Table 6:** Chemical composition analysis using EDX (n.d. = not detected).

Oxides (%)	CSH Gels	Ettringite
MgO	12.74 ± 2.35	n.d.
Al_2_O_3_	6.65 ± 2.19	16.57 ± 2.25
SiO_2_	20.91 ± 2.86	6.26 ± 2.87
SO_3_	4.25 ± 1.63	22.71 ± 4.72
P_2_O_5_	3.34 ± 1.25	n.d.
K_2_O	0.88 ± 0.32	n.d.
CaO	48.80 ± 4.26	54.43 ± 4.53
Fe_2_O_3_	2.44 ± 0.71	n.d.

**Table 7:** Chemical composition (%) of the materials.

Oxides (%)	OPC	10% MA	20% MA	EN 197-1
SiO_2_	20.06	18.32	16.57	-
Al_2_O_3_	4.96	4.57	4.17	-
Fe_2_O_3_	3.28	3.03	2.78	-
CaO	62.19	60.51	58.84	-
MgO	2.12	2.54	2.97	-
SO_3_	3.14	3.24	3.34	≤3.5–4.0
Na_2_O	0.37	0.38	0.39	-
K_2_O	0.66	1.29	1.91	-
TiO_2_	0.25	0.23	0.22	-
P_2_O_5_	0.29	0.37	0.46	-
Cl	0.06	0.05	0.05	≤0.1
ZnO	0.17	0.15	0.14	
SrO	0.16	0.14	0.13	
Cr_2_O_3_	0.04	0.04	0.03	
LOI	2.11	4.90	7.69	

**Table 8:** Rheological behavior of the blended cement pastes.

	OPC	10% MA	20% MA	EN Standard
NCW (±1 g)	150	151	154	-
IST (±10 min)	138	129	126	≥60
S (mm)	0.15	0.10	0.10	≤10

**Table 9:** Heat of hydration (J/g) at 41 h of reaction.

Sample	Time (h)	Heat of Hydration (J/g)
OPC	41	317.4
10% MA	41	285.54
20% MA	41	265.54

**Table 10:** Absorption coefficients in mortars.

Cement	Total Water Absorption (wt%)	Absorption Rate (g/min^0.5^)	R^2^
OPC	4.26	0.78	0.96
10% MA	4.83	0.86	0.97
20% MA	5.63	0.94	0.97

**Table 11:** Water absorption rates (g/s).

Material	Rate 1	Rate 2	Rate 3
Intervals	2.24–7.75 min^0.5^	10.95–18.97 min^0.5^	37.95–14,400 min^0.5^
OPC	0.682	0.058	0.006
10% MA	0.729	0.067	0.005
20% MA	0.788	0.092	0.007

**Table 12:** K, $\varepsilon_{e}$, and m-coefficients.

Material	K (kg/m^2^min^0.5^)	$\varepsilon_{e}$ (cm^3^/cm^3^)	m (min/cm^2^)
OPC	0.736	0.788	87.210
10% MA	1.112	1.190	87.316
20% MA	1.173	1.252	87.698

**Table 13:** Values for the q, $\rho_{0}$ and R^2^ parameters of the mortars studied.

	OPC	10% MA	20% MA
q	0.231	0.246	0.265
$\rho_{0}$	19.716	17.566	15.179
R^2^	0.964	0.969	0.971

**Table 14:** Compressive strength (MPa) of the mortars and mechanical requirements.

Mortar	2 d	28 d	90 d	EN 197-1
OPC	41.23 ± 0.01	67.17 ± 0.18	69.03 ± 1.07	≥20–30 (2 d) ≥52.5 (28 d)
10% MA	34.73 ± 0.24	59.29 ± 0.16	60.50 ± 0.08	
20% MA	29.27 ± 0.46	50.50 ± 0.61	52.81 ± 0.05	

**Table 15:** Total mortar porosity (vol. %) at 2 and 90 d.

	Total Porosity (vol. %)	
	2 d	90 d
OPC	13.96	11.70
10% MA	16.83	13.01
20% MA	17.14	14.20

## Data Availability

The raw data supporting the conclusions of this article will be made available by the authors on request.
